# Colorectal cancer risk assessment and screening recommendation: a community survey of healthcare providers' practice from a patient perspective

**DOI:** 10.1186/1471-2296-13-17

**Published:** 2012-03-14

**Authors:** Ryan J Courtney, Christine L Paul, Robert W Sanson-Fisher, Finlay A Macrae, Mariko L Carey, John Attia, Mark McEvoy

**Affiliations:** 1 The Priority Research Centre for Health Behaviour, School of Medicine and Public Health, Faculty of Health, The University of Newcastle, Newcastle, Australia; 2 Department of Colorectal Medicine and Genetics, The Royal Melbourne Hospital, Newcastle, Australia; 3 The Centre for Clinical Epidemiology and Biostatistics, Faculty of Health, The University of Newcastle, Newcastle, Australia; 4 Hunter Medical Research Institute, Newcastle, Australia

## Abstract

**Background:**

Family history is a common risk factor for colorectal cancer (CRC), yet it is often underused to guide risk assessment and the provision of risk-appropriate CRC screening recommendation. The aim of this study was to identify from a patient perspective health care providers' current practice relating to: (i) assessment of family history of CRC; (ii) notification of "increased risk" to patients at "moderately/potentially high" familial risk; and (iii) recommendation that patients undertake CRC screening.

**Methods:**

1592 persons aged 56-88 years randomly selected from the Hunter Community Study (HCS), New South Wales, Australia were mailed a questionnaire. 1117 participants (70%) returned a questionnaire.

**Results:**

Thirty eight percent of respondents reported ever being asked about their family history of CRC. Ever discussing family history of CRC with a health care provider was significantly more likely to occur for persons with a higher level of education, who had ever received screening advice and with a lower physical component summary score. Fifty one percent of persons at "moderately/potentially high risk" were notified of their "increased risk" of developing CRC. Thirty one percent of persons across each level of risk had ever received CRC screening advice from a health care provider. Screening advice provision was significantly more likely to occur for persons who had ever discussed their family history of CRC with a health care provider and who were at "moderately/potentially high risk".

**Conclusions:**

Effective interventions that integrate both the assessment and notification of familial risk of CRC to the wider population are needed. Systematic and cost-effective mechanisms that facilitate family history collection, risk assessment and provision of screening advice within the primary health care setting are required.

## Background

### Colorectal cancer (CRC) screening: reduces the burden of disease

Worldwide, CRC comprises 9.4% of all cancer cases with one million diagnoses annually [1]. Survival from CRC is highly stage-dependent yet fewer than 40% of CRCs are diagnosed at a localised stage [2]. Screening is a cost-effective mechanism that reduces the incidence and mortality associated with CRC [3-5]. Population-based screening is recommended for those aged 50 years or older, with average-risk persons recommended to receive periodical Faecal Occult Blood Test (FOBT) screening or endoscopy screening, dependent on screening guidelines [6,7]. Several randomised controlled trials (RCTs) have indicated it is possible to reduce mortality from CRC mortality by 15% to 33% with Faecal Occult Blood Test (FOBT) screening on either an annual or biennial basis[3,4,8-10]. Recent RCT evidence of once-only flexible sigmoidoscopy for persons 55-64 years of age provides strong evidence for the substantial and long-term benefit of this procedure [11]. This study indicated a one-third reduction of incidence and 40% reduction in mortality for persons undertaking sigmoidoscopy screening [11]. Although colonoscopy remains untested in randomised trials, case control and cohort studies using large-bowel endoscopy have shown a CRC mortality reduction rate ranging from 60% to 76% [12] and incidence reduction of 76% to 90% [5].

### Family history is a common and important risk factor

Relatives of CRC patients are a group that can benefit from targeted screening or prevention programs [13]. Approximately 15% to 20% of CRC cases occur in persons who have a first degree relative with the disease [14-16]. Relative risk is up to two-fold for persons with a first-degree relative diagnosed with CRC and three- to six- fold for families with additional factors such as early onset of CRC or multiple relatives affected [6,17]. The presence of genetically-based familial syndromes, such as hereditary non-polyposis CRC (HNPCC) and familial adenomatous polyposis (FAP) further increases the risk of CRC in first-degree relatives [7,14]. Screening guidelines in Australia categorise asymptomatic individuals into three levels of risk: "at or slightly above average risk"; "moderately increased risk'; and "potentially high risk". Risk is quantified based on family history of CRC: number and type of relative diagnosed (first or second-degree relative) and their age at diagnosis, with additional high-risk features considered [6].

### CRC risk assessment in the primary care setting

Health care providers play an integral role in cancer control by identifying those individuals whose behaviour, environment or family history place them at increased risk of CRC [18].

In Australia, CRC screening guidelines entrust healthcare providers with preventive practices relating to: patient family history of CRC assessment; the notification of persons at increased-risk; and the provision of risk-appropriate CRC screening advice [6]. However, evidence suggests that family-health histories are seldom taken in primary-care [19]. This may be due to the competing demands for care and time factors that are placed on general practitioners [20,21]. In addition, the complexity of familial-risk interpretation and the lack of systematic collection and assessments methods to assist primary-care providers further compound this problem [19]. Retrospective chart audits have demonstrated that 40% to 55% of patient medical records include important family history information (i.e. the presence or absence of breast or bowel cancer) [22,23]. Clinicians' self reported rate of patient family history assessment ranges from 30% to 90% [24,25]. Direct observation studies suggest discussion of family history may occur in 51% of visits by new patients and 22% of visits with established patients [26]. However, relatively little is known about current practice relating to health care providers' identification and notification of persons at elevated-risk of CRC. The few studies [18,23,27,28] which have assessed this issue have been conducted within North American family practice settings adopting patient record audit.

### The opportunity often missed: CRC screening recommendation to patients

Medical practitioner recommendation can increase the likelihood of complying with CRC screening up to 23-times [29,30], with over 75% of persons reporting that they would undertake screening if it were recommended by a doctor [31,32]. Nonetheless, CRC screening recommendations are neither routinely nor consistently provided to at-risk patients [33-36]. Population-based data from the United States National Health Interview Survey (NHIS) indicated that only 10% of under-screened patients who had visited their doctor in the previous year had received a CRC screening recommendation [35]. In Australia, a recent evaluation of at-risk persons (aged 30-70 years) presenting to an out-patient hospital setting identified that 22% of persons had ever received any CRC screening recommendation from their medical practitioner [37].

To our knowledge the current study presents as the first community-based evaluation of health care providers practice relating to CRC family history assessment and notification of "increased risk" to persons at elevated levels of familial risk ("moderately increased/potentially high risk"). Further, the socio-demographic, lifestyle or psychosocial factors associated with family history assessment of CRC and provision of screening advice outside of the general practice setting is understudied [35]. Both the identification of these factors and sub-groups less likely to receive CRC family history assessment and screening advice is of importance for the development and improvement of future preventive programs in the general practice setting. The aim of this study was to examine among a community-based cohort of at-risk persons (aged 56-88 years), the proportion of respondents:

(i) Who had ever been asked by a health care provider about their family history of CRC

(ii) At elevated levels of familial risk ("moderately increased/potentially high risk") who had been notified by a health care provider of their "increased risk" of developing CRC.

(iii) Who had ever received CRC screening advice from a health care provider.

Socio-demographic, clinical and psychosocial characteristics associated with ever asked about family history of CRC and ever received CRC screening advice were identified.

## Methods

### Design and study population

The Hunter Community Study (HCS) sample is a longitudinal cohort of community dwelling men and women aged 55-85 years at baseline in the Hunter Region, NSW, Australia [38]. Participants were randomly selected from the NSW State electoral roll between December 2004 and 2007. The HCS cohort reflects the Hunter Region, state and national profiles for gender and marital status, but is slightly younger in age [38]. A randomly selected sub-sample of HCS participants (n = 1592) aged 56-88 years at time of survey (November, 2009) were mailed a pen and paper questionnaire.

### Questionnaire

Respondents were asked about health care providers' practice relating to assessment of family history of CRC - "Has any health professional e.g. your doctor, ever asked if you have a family history of bowel cancer" (Yes/No), notification of "increased risk" of developing CRC - "Did this person discuss whether there was a possible 'increased risk' of developing bowel cancer for you or your family members?" (Yes/No), and the provision of CRC screening information - "Has any health professional suggested that you or your relatives should do any of the following: Please choose all that apply." (i. Start having screening tests for bowel cancer/ii. Talk to their doctor about screening tests/iii. No advice has ever been given/iv. Take other action related to bowel cancer (please specify___). Responses to questions relating to ever asked about family history of CRC by a health care provider and notification of possible increased risk were used to derive a *Discussion of family history of CRC with doctor *variable with three levels (never discussed, discussed and informed of possible "increased risk", discussed and not informed of possible "increased risk"). Respondents' answers concerning provision of CRC screening information were used to derive an *ever received screening advice from doctor *variable (Yes/No). Respondents indicating (i), (ii), or (iv) were coded as ("Yes") to receiving screening advice while respondents answering (iii) were coded as ("No").

### Respondents familial risk based on self reported family history of CRC

Respondents were asked separately whether any first- or second- degree relatives had ever been diagnosed with CRC and if so, their age at diagnosis. Survey items used to assess family history of CRC are presented in Additional file 1: Appendix A. Respondents' self reported family history of CRC was used to allocate persons to their level of risk in accordance with current clinical practice guidelines (see Table 1).

**Table 1:** Criteria used to define risk categories in accordance with national CRC screening guidelines.

Risk Category*	
At or slightly above average risk	• No personal history of bowel cancer. • Either no close relatives with bowel cancer or one first-degree or second-degree relative with bowel cancer diagnosed at age 55 years or older.

Moderately increased risk	• One first-degree relative diagnosed before the age of 55 years (without potentially high-risk features listed below), or • Two first-degree relatives or one first- and one second-degree relative(s) on the same side of the family (without potentially high-risk features listed below).

Potentially high risk	• Three or more first-degree or a combination of first-degree and second-degree relatives on the same side of the family diagnosed with bowel cancer (suspected HNPCC**), or Two or more first-degree or second-degree relatives on the same side of the family diagnosed with bowel cancer, including the following risk feature: bowel cancer before the age of 50 years or at least one-relative with cancer of the endometrium, ovary, stomach, small bowel, renal pelvis, ureter, biliary tract or brain.

* Risk features potentially placing persons at possible increased risk, including personal history of adenoma, inflammatory bowel disease or suspected familial adenomatous polyposis (FAP) were not assessed in the survey. **HNPCC (Hereditary non-polyposis colorectal cancer), also known as Lynch syndrome.

### Statistical Analysis

Frequency distributions (n/%) with 95% confidence interval were used to assess the proportion of respondents that had ever been asked about their family history of CRC by a health care provider (Yes/No) and ever received CRC screening advice (Yes/No). For persons at elevated level of risk ("moderately increased/potentially high risk") the proportion notified of their "increased risk" was identified. The following items selected from the HCS databank were assessed as potential correlates of ever asked about family history of CRC and ever received CRC screening advice: *Socio-demographic and lifestyle*, i.e. age, gender, education, marital status, country of birth, household income, retirement, private health insurance status, tobacco or alcohol use; *Clinical*, i.e. general practice visits per year, previous cancer diagnosis (excluding CRC), body mass index, and co-morbidity (e.g. high cholesterol, hypertension, asthma, diabetes); and *psychosocial*, i.e. physical health, assessed using the physical health component summary score (PCS) on the short form health survey SF-36 [39], and mental health assessed using the Kessler Psychological Distress Scale (K-10) [40]. The PCS is a physical health summary score aggregated from the physical functioning, role-physical, bodily pain and general-health scales on the SF-36 [41]. Multiple logistic regression analysis was used to determine independent factors associated with ever asked about family history of CRC by a health care provider and ever received CRC screening advice. Variables with a *p *value < .25 following simple logistic regression analysis (See Additional file 1: Appendix B) were entered into multiple logistic regression model (with both forward and backward stepwise elimination used to check consistency of results). Variables that met the significance cut-point of (*p *value < .05) were entered into the final model. Data were analysed using STATA 11 (STATA, Texas, USA).

### Ethics approval

The University of Newcastle and Hunter New England Population Health Human Research Ethics Committees granted ethical approval.

## Results

### Characteristics of the sample

Of the 1592 mailed surveys, 1117 respondents completed and returned surveys (response rate, 70%). Respondents previously diagnosed with CRC (n = 24) or reporting they had undergone major abdominal surgery (n = 8) were excluded from analysis, leaving a total sample of 1085 eligible participants with data. 1061 participants provided adequate family history information for the purposes of risk allocation. Socio-demographic characteristics and the proportion of respondents at each level of risk are presented in Table 2.

**Table 2:** Socio-demographic characteristics of study respondents (n = 1085)

Characteristic	n	%
Gender		

Male	508	47

Female	577	53

Age (years)		

56-64	455	42

65-74	382	36

75-88	237	22

Country of Birth		

Australia	885	89

Other	111	11

Marital status		

In a relationship	805	77

Not in relationship	240	23

Risk category		

At or slightly above average risk	979	92

Moderately increased risk	52	5

Potentially high risk	30	3

Annual household income before tax ($)		

< = 39, 999	574	58

40, 000 - 69, 999	216	22

> = 70,000	197	20

Highest Level of Education		

Secondary schooling (not-completed)	229	22

Secondary schooling (completed)	241	23

Trade qualification or TAFE:	264	25

University or other tertiary study	256	25

Other or not applicable	53	5

* Percentage of responses (excluding any missing values).

### Family history of CRC assessment

1050 respondents provided information on "ever asked about family history of CRC by a health care provider" (Yes/No). Overall 38.3% (95% CI, 35.3 - 41.3) of respondents had ever been asked if they had a family history of CRC by a health care provider. Risk category specific rates were as follows: "at or slightly above average risk" (36.6%, 33.6 - 39.7), "moderately increased risk" (54.9%, 40.3 - 68.9) and "potentially high risk" (65.6%, 45.7 - 82.1). Table 3 presents the results of the multiple logistic regression analysis. Persons with a higher education (university or other tertiary study), ever receiving screening advice from a health care provider and with lower physical component summary (PCS) score on the SF-36 were significantly more likely to have ever had been asked about their family history of CRC by a health care provider.

**Table 3:** Multiple logistic regression analysis (n = 870)* of factors associated with ever asked about family history of CRC by a health care provider

	Asked by health care provider n (%)	OR (95%CI)	*p*- value
Education			

Secondary schooling (not-completed)	61 (34)	1	

Secondary schooling (completed)	67 (33)	.99 (.61, 1.62)	.974

Trade qualification or TAFE:	83 (38)	1.24 (.77, 2.00)	.373

University or other tertiary study	108 (48)	2.14 (1.34, 3.45)	.002

Other or not applicable	19 (44)	1.89 (.88, 4.05)	.102

Ever received screening advice from			

doctor			

Yes	206 (73)	10.11 (7.24, 14.12)	.000

No	132 (22)	1	

SF-36 (PCS)	-	.98 (.96, .99)	.012

* Respondents excluded from model due to missing values (n = 191)

### Health care providers' practice relating to notification of "increased risk" to persons at elevated levels of risk ("moderately increased/potentially high risk")

Of the 82 respondents classified at "moderately increased risk" or "potentially high risk", 78 persons provided information relating to "discussion of family history of CRC with doctor". 42.3% (95% CI 31.2 - 54.0) of such persons had never been asked by a health care provider about their family history of CRC. 51.3% (39.7 - 62.8) had been both asked about their family history of CRC and notified of possible "increased risk". A further 6.4% (2.1-14.3) had been asked about their family history of CRC but were not informed of any possible "increased risk".

### Health care providers' practice relating to CRC screening recommendation

Across the entire cohort 978 respondents provided information on CRC screening advice. Overall, 31.1% (95% CI 28.2 - 34.1) of respondents had ever received screening advice from a health care provider. Risk-specific rates of receiving screening advice for persons were: 27.7% (24.8 - 30.7) for those "at or slightly above average risk", 67.3% (24.8-30.7) for those at "moderately increased risk" and 80.8% (60.6-93.4) for those at "potentially high risk". Multiple logistic regression analysis (See Table 4) found that persons: at elevated level of risk ("moderately increased/potentially high risk") and who had ever discussed family history of CRC with doctor irrespective of whether informed of "increased risk" were significantly more likely to ever had received screening advice from a health care provider.

**Table 4:** Multiple logistic regression analysis (n = 956)* of factors associated with ever receiving CRC screening advice from a health care provider

	Received screening advice n (%)	OR (95%CI)*	*p*-value
Risk Category			

At or slightly above average risk	239 (27)	1	

Moderately increased risk	32 (68)	4.45 (2.07, 9.55)	.000

Potentially high risk	20 (80)	6.92 (2.20, 21.71)	.001

Discussion of family history of CRC with doctor			

Never discussed	81 (13)	1	

Discussed/informed of 'increased' 'risk'	152 (76)	17.96 (11.97, 26.95)	.000

Discussed/not informed of increased 'risk'	58 (38)	4.12 (2.73, 6.20)	.000

* Respondents excluded from model due to missing values (n = 105)

### Risk notification and CRC screening recommendation for persons at elevated levels of risk ("moderately increased/potentially high risk")

Of the 82 respondents at either 'moderately increased risk' or 'potentially high risk' 72 persons provided information on both 'discussion of family history of CRC with doctor' and 'ever received CRC screening advice'. For respondents at each respective level of risk; 95% (38/40) of persons notified of their 'increased risk' of CRC had ever received screening advice compared to 37% (10/27) of respondents who had never discussed their family history of CRC with a doctor. Eighty per cent (4/5) of persons who had discussed their family history of CRC with a doctor but were not informed of their 'increased risk' had ever received CRC screening advice.

## Discussion

Study findings indicate that opportunities for earlier detection of CRC are being missed, with 38% of all respondents ever asked about their family history of CRC by a health care provider. Of concern is that close to half of persons at "moderately increased/potentially high risk" had not been notified of their "increased risk" by a health care provider. Overall, the rate of screening advice was low with approximately one-third of respondents irrespective of risk category ever receiving CRC screening advice from a health care provider.

### Risk-based colorectal cancer screening

Before further consideration of study findings, the importance of "risk-based" screening and some constraints of population-level CRC screening must be addressed. Population-level screening has demonstrated effectiveness with RCTs indicating a CRC mortality reduction between 15 to 33% [3,4,8-10], with a slightly higher benefit secured from sigmoidoscopic screening [11]. At present, there is still no RCT evidence for colonoscopy screening. While population-level screening indicates efficacy for the "average risk" population it does not cater for the tailored screening requirements of higher risk groups. Risk-based screening, as adopted in the Australian clinical practice guidelines [6], is an attempt to address the critical relationship between strong family history of CRC and the increased likelihood of developing the disease. This risk-based approach to screening, also acknowledges the problem of over screening, which can be harmful, costly and unnecessary. While it is pertinent all patients receive CRC screening advice from their healthcare provider, it is critical that family history of cancer discussions guide risk-appropriate CRC screening provision to patients. Correct risk assignment and clinical management is of critical importance for patients with family histories suggestive of hereditary cancer syndromes.

### Family history assessment in the primary care setting

Family history assessment is important for identification of those who may benefit from tailored screening or referral to genetic services. This study suggests that the important first-step in identification of risk - discussion of family history of CRC- often does not occur. Such findings are consistent with previous data identifying poor documentation of family history information in the family practice setting [18,23,26,28,42]. It must be acknowledged that family history assessment and notification of risk does not ensure continued adherence to screening recommendations [43]. Nonetheless, risk communication is an essential prerequisite to patients' understanding of their risk. Current study findings suggest the need for strategies that help to improve the assessment of family history of CRC and the identification of potential familial risk in the primary care-setting

### Improving family history assessment and referral

Health care providers report the need for clear guidance on the risk stratification and referral process of patients [44,45]. Further refinement of user-friendly guidelines that help to facilitate identification and referral of increased-risk patients is an important yet necessary step forward as suggested by other researchers [18,42,44,46]. The cumbersome and time-consuming nature of family history assessment has been a barrier to accurate assessment of familial risk [46-48], which may in part be overcome by the advent of computerised Cancer Risk Assessment Tools (CRATs) which have helped to standardise and simplify family history assessment [49,50]. Interventions using this technology designed to efficiently assess and automate risk stratification are likely to increase the utility of family history assessment in the primary health care setting [51]. Recent cluster randomised controlled trials have demonstrated this tool's effectiveness in the management of familial breast and CRC including a trial which compared a computer decision support aid versus best evidence practice (education and guideline dissemination) [19,52].

### Improving the CRC screening message to the at-risk population

Current findings indicate the need for practice-based strategies that systematically prompt health care providers to encourage CRC screening among at-risk patients, especially given patients are often reluctant to initiate discussions [53]. Little data exists on how often CRC screening is included in office reminder systems and the reasons for under utilisation in the primary health care setting [53]. Although meta-analyses have demonstrated that minimal prompts to patients and providers are effective in enhancing CRC screening with FOBT[54], research suggests very limited use of such reminder systems for CRC screening [54,55]. Intuitively maximising the wider adoption of simple systematic practice-based interventions that include prompts to health care providers for CRC screening may assist in elevating CRC screening rates across the at-risk population.

### Study limitations

The findings should be interpreted in light of some methodological limitations. Health care providers' practices relating to preventive measures in this study were based on self-report with associated potential for recall bias. Self-reported family history of CRC was not validated against objective sources, a limitation however that is consistent with other published studies that have used patient record audit [18,23,28]. For a small minority of respondents at "moderately increased/potentially high risk", the level of risk may have been overestimated. For respondents indicating that both a first- and second-degree relative were diagnosed with CRC, an assumption was made that both relatives were diagnosed on the same side of the family. Participants' report of health care providers' assessment of patient family history of CRC may have been underestimated due to several factors including recall bias. It is possible that health care providers may have asked a broader question about family history of cancer or respondents could have instigated family history discussions without health care providers' initiation. However, the literature suggests such patient initiation is rare[53]. Alternatively, health care providers may have been aware of a persons' familial risk from information obtained previously from other relatives in the family with notification of "increased risk" given on this basis. Further, it is important to consider that for some persons their risk-status may have changed over-time (since first discussion of family history with a doctor). That is, some persons asked about their family history of CRC (years prior to survey completion) may have had no family history of CRC at this time and were correctly notified of no "increased risk", yet at a later stage, following a relative's diagnosis of CRC were notified by a doctor of their "increased risk". While this may have occurred in a small number of persons, the literature suggests that periodic updating of family history of CRC information is seldom conducted in the primary-care setting [22,26,56]. Subsequently, it is highly possible that for the most part, persons with a changed family history of CRC overtime would not have been notified of their changed risk status. Finally, some participants (recently turning 55 or 65 years of age) may have had recent contact with the National Bowel Cancer Screening Program prior to survey completion. As part of this program, an FOBT screening invitation and information on familial risk and CRC screening is provided, which may have increased CRC awareness in this particular respondent group.

## Conclusions

The current study's evaluation of patient self-report suggests that family history of CRC discussions with a health care provider had occurred in a minority of cases. Consequently, many families at the greatest risk of developing CRC may have missed a vital opportunity to gain benefits from increased risk notification, risk-appropriate screening and adequate referral to genetic consultation. Greater emphasis on the refinement and development of user-friendly referral guidelines, the wider incorporation of office-based reminder and computer-based technology to facilitate risk assessment and CRC screening recommendation is required.

## Competing interests

The authors declare that they have no competing interests.

## Authors' contributions

RJC, CLP, RSF were responsible for study design, data collection, analysis and drafting. All authors contributed to the interpretation of results and writing of this manuscript.

## Funding

The project was funded by the Lion's Club of Adamstown in conjunction with the Hunter Medical Research Institute.

## Pre-publication history

The pre-publication history for this paper can be accessed here:

http://www.biomedcentral.com/1471-2296/13/17/prepub

## Supplementary Material

Additional file 1 **Appendix A**. Items and response options (verbatim) used to assess respondents family history. Appendix B. Simple logistic regression analyses of factors associated with ever asked about family history of CRC by a healthcare provider and ever receiving CRC screening advice. Click here for file
