# Supplementary material for: Colorectal cancer risk assessment and screening recommendation: a community survey of healthcare providers' practice from a patient perspective
Source: BMC Fam Pract. 2012 Mar 14;13:17. doi: 10.1186/1471-2296-13-17 (PMC3323420; doi:10.1186/1471-2296-13-17)
Supplement: Additional file 1 — Appendix A. Items and response options (verbatim) used to assess respondents family history. Appendix B. Simple logistic regression analyses of factors associated with ever asked about family history of CRC by a healthcare provider and ever receiving CRC screening advice. [file 1471-2296-13-17-S1.DOC]

**Appendix A**

Items and response options (verbatim) used to assess respondents family history*.

| First-degree relatives.  “How many of your close relatives (mother, father, brother, sister or child) have ever been diagnosed with bowel cancer?”  🞎 None  🞎 One  🞎 Two  🞎 Three or more |
| --- |
| Second-degree relatives.  “Have any of your second-degree relatives (grandparents, uncles, aunts, nephews, nieces or half-siblings) ever been diagnosed with bowel cancer? This includes only biological/blood relatives not those related to you through marriage. Please select all that apply”.  🞎 None of these relatives have ever been diagnosed with bowel cancer  🞎 Yes, mother’s mother  🞎 Yes, father’s mother  🞎 Yes, mother’s father  🞎 Yes, father’s father  🞎 Yes, mother’s sister  🞎 Yes, father’s sister  🞎 Yes, mother’s brother  🞎 Yes, father’s brother  🞎 Yes, nephew  🞎 Yes, niece  🞎 Yes, grandchild  🞎 Yes, half-sibling (mother’s side)  🞎 Yes, half sibling (father’s side) |
| Age at diagnosis (asked separately for both first-and second degree relatives).  How many of these relative(s) were diagnosed at the following ages? (Please write number of relatives diagnosed in each age category)  __ 50 or less  __ Between 51 and 55  __ 56 or over  __ Don’t know |
| Other cancers (high-risk feature).  Has your mother, father, brother, sister, child, grandparent, uncle, aunt, nephew, niece, or half-sibling ever been diagnosed with any of the following cancers: endometrium, ovary, stomach, renal pelvis, ureter, biliary tract or brain.  🞎 Yes  🞎 No  🞎 Don’t know |

**Appendix B**

Simple logistic regression analyses of factors associated with ever asked about family history of CRC by a healthcare provider and ever receiving CRC screening advice.

|  | Ever asked about family history of CRC | | CRC screening advice | |
| --- | --- | --- | --- | --- |
|  | OR (95%CI) | *p*-value | OR (95%CI) | *p*-value |
| *Socio-demographic characteristics* |  |  |  |  |
| Gender |  |  |  |  |
| Female | 1 |  | 1 |  |
| Male | .91 (.71, 1.17) | .467 | 1.07 (.81, 1.39) | .639 |
| Age (years) |  |  |  |  |
| 56-64 | 1 |  | 1 |  |
| 65-74 | 1.02 (.77, 1.35) | .905 | 1.11 (.82, 1.50) | .497 |
| 75-88 | .62 (.44, .87) | **.006** | .65 (.44, .95) | **.026** |
| Marital status |  |  |  |  |
| Married/ Living with partner | 1 |  |  | 1 |
| Never married/ Widowed/ Divorced or separated | .97 (.72, 1.32) | .866 | 1.00 (.72, 1.40) | .971 |
| Education |  |  |  |  |
| Secondary schooling (not-completed) | 1 |  | 1 |  |
| Secondary schooling (completed) | .99 (.68, 1.47) | .990 | 1.02 (.67, 1.54) | .926 |
| Trade qualification or TAFE: | 1.14 (.78, 1.66) | .501 | 1.07 (.71, 1.59) | .749 |
| University or other tertiary study | 1.64 (1.13, 2.38) | **.010** | 1.25 (.84, 1.86) | .280 |
| Other or not applicable | 1.34 (.72, 2.49) | .363 | .87 (.43, 1.76) | .700 |
| Household income before tax ($) |  |  |  |  |
| <= 39, 999 | 1 |  | 1 |  |
| 40, 000 – 69, 999 | 1.01 (.73, 1.41) | .934 | 1.07 (.76, 1.52) | .698 |
| >= 70,000 | 1.47 (1.05, 2.04) | **.023** | 1.23 (.86, 1.75) | **.248** |
| Country of birth |  |  |  |  |
| Australia | 1 |  | 1 |  |
| Other | .64 (.41, .99) | **.049** | .60 (.36, .98) | **.041** |
| Retired |  |  |  |  |
| Yes | .87 (.67, 1.12) | .275 | 1.04 (.79, 1.38) | .779 |
| No | 1 |  | 1 |  |
| Private health insurance |  |  |  |  |
| No coverage | 1 |  | 1 |  |
| Coverage | 1.43 (1.06, 1.93) | **.019** | 1.51 (1.09, 2.09) | **.014** |
| Alcohol |  |  |  |  |
| Drink days per month | 1.00 (1.06, 1.93) | .555 | 1.00 (.99, 1.02) | .590 |
| Smoke |  |  |  |  |
| Never | 1 |  | 1 |  |
| Ever | 1.13 (.87, 1.48) | .358 | 1.13 (.85, 1.50) | .401 |
| Now | .62 (.36, 1.06) | **.082** | .74 (.42, 1.29) | .282 |
| *Clinical characteristics* |  |  |  |  |
| GP visits per year |  |  |  |  |
| None to twice | 1 |  | 1 |  |
| Three to six | 1.09 (.80, 1.49) | .590 | 1.40 (.99, 1.98) | **.056** |
| > six | 1.23 (.85, 1.79) | .275 | 1.33 (.87, 2.02) | **.189** |
| Previous Cancer (excluding CRC) |  |  |  |  |
| Yes | .80 (.59, 1.10) | **.172** | 1.03 (.74, 1.44) | .865 |
| No | 1 |  | 1 |  |
| Risk category |  |  |  |  |
| At or slightly above average risk | 1 |  | 1 |  |
| Moderately increased risk | 2.11 (1.20, 3.72) | **.010** | 5.39 (2.91, 9.96) | **.000** |
| Potentially high risk | 3.29 (1.51, 7.16) | **.003** | 10.97 (4.09, 29.41) | **.000** |
| Discussion of family history of CRC with doctor |  |  |  |  |
| Never discussed | NA |  | 1 |  |
| Discussed, informed of ‘increased risk’ |  |  | 19.91 (13.44, 29.51) | **.000** |
| Discussed, not informed of ‘increased risk’ |  |  | 3.79 (2.54, 5.65) | **.000** |
| Screening advice ever provided by health care provider |  |  |  |  |
| Yes | 9.40 (6.87, 12.84) | **.000** | NA |  |
| No | 1 |  |  |  |
| BMI |  |  |  |  |
| < 18.5 | .25 (.03, 2.04) | **.193** | .54 (.11, 2.66) | .445 |
| 18.5 - 25 | 1 |  | 1 |  |
| > 25 | 1.12 (.81, 1.56) | .498 | .85 (.61, 1.20) | .350 |
| Comorbidity |  |  |  |  |
| Yes | 1.36 (.99, 1.87) | **.060** | 1.29 (.92, 1.82) | **.140** |
| No | 1 |  | 1 |  |
| *Psychosocial characteristics* |  |  |  |  |
| SF-36 (physical health component score) | .99 (.97, 1.00) | **.074** | 1.00 (.99, 1.02) | .595 |
| K-10  (mental health) |  |  |  |  |
| Low or no risk (10-15) | 1 |  | 1 |  |
| Medium to high risk (16 +) | .93 (.70, 1.23) | .603 | .85 (.63, 1.16) | .316 |

* *p*-values <.25 included in multiple regression model bold
